# Soil microbial necromass carbon contributions to soil organic carbon after three decades of citrus cultivation

**DOI:** 10.3389/fmicb.2025.1589966

**Published:** 2025-05-14

**Authors:** Tangyingze Mei, Quanchao Zeng, Ruifeng Chen, Wenfeng Tan

**Affiliations:** ^1^College of Resources and Environment, Huazhong Agricultural University, Wuhan, China; ^2^Chongqing Institute of Green and Intelligent Technology, Chinese Academy of Sciences, Chongqing, China

**Keywords:** soil organic carbon, citrus planted years, microbial-associated organic carbon, microbial necromass carbon, forest

## Abstract

**Introduction:**

Citrus is one of the most economically significant fruits globally, and soil organic carbon (SOC) plays a central role in maintaining soil health and fertility. Consequently, enhancing SOC content directly influences both the yield and quality of citrus crops. However, the sources of SOC in citrus orchards and their mechanisms of contribution remains poorly understood.

**Methods:**

This study investigated citrus soils from orchards of varying planting ages by collecting 0–20 cm soil samples. We analyzed amino sugars, glomalin, particulate organic carbon (POC), and mineral-bound organic carbon (MAOC) to examine the source of microbial residue carbon and its contribution to SOC.

**Results:**

The results revealed a significant decrease in microbial residue carbon (MNC), fungal residue carbon (FNC), and bacterial residue carbon (BNC) with increasing orchard age (*p* < 0.05). Specifically, the MNC in 30-year-old citrus soils was reduced by 46.27% compared to 10-year-old soils, FNC decreased by 45.61%, and BNC by 48.91%. The proportion of microbial residue carbon within SOC significantly decreased as planting years increased (*p* < 0.05), from 76.82 ± 2.84% in 10-year-old citrus soils to 20.54 ± 4.70% in 30-year-old soils. Furthermore, soil pH, NO₃^−^-N and MAOC were the main factors controlling MNC. MNC showed a significant negative correlation with SOC, indicating a weakened microbial carbon pump function in citrus soils and an increased reliance on other carbon sources, such as plant-derived carbon. Although citrus cultivation had led to a decline in microbial residue carbon over time, it remained a primary source of organic carbon, with its contribution depending on the age of the orchard.

**Discussion:**

These findings offered novel insights into the mechanisms through which intensive citrus cultivation influences microbial necromass contributions to SOC. This study also highlighted the negative impacts of long-term citrus cultivation on soil microbial necromass and offered recommendations for the rehabilitation of aging orchards.

## 1 Introduction

Soil organic carbon (SOC) is a core element of soil health and fertility, directly influencing soil structure, nutrient cycling, and plant growth (Hartmann and Six, 2023). It plays a crucial role in agricultural ecosystems, particularly in the production of economic crops such as citrus. Citrus is one of the most important fruit crops worldwide, and as a major agricultural product, it not only holds significant value for food production but also makes a substantial contribution to the agricultural economy. However, with the ongoing development of citrus cultivation, especially the changes in soil environment from long-term planting, the trends and mechanisms underlying SOC variation remain unclear. Understanding the sources and turnover mechanisms of SOC is crucial for improving citrus yield and quality and for protecting soil resources, making it of both theoretical significance and practical value for optimizing soil management practices and enhancing the sustainability of citrus production.

Soil microbial communities play a critical role in the turnover of SOC, as microbes decompose plant residues and soil organic matter, facilitating the mineralization and transformation of organic carbon (Kallenbach et al., 2015; Kästner et al., 2021; Camenzind et al., 2023). Meanwhile, microorganisms assimilate labile organic matter into biomass through anabolic processes, which, upon death, contribute to the long-term stabilization of necromass in the soil (Liang et al., 2017; Liang et al., 2020). Soil microbial residue carbon (such as bacterial and fungal residue carbon) is an important component of SOC, playing a key role in the storage and release of organic carbon (Kästner et al., 2021; Wang et al., 2023). It has been reported that soil microbial necromass carbon (C) contributes 51% to the 0–20 cm SOC in global farmland soils (Wang B. et al., 2021), underscoring the dominant role of soil microorganisms in SOC dynamics. Therefore, understanding the sources, composition, and influencing factors of soil microbial necromass is essential for effectively managing SOC in agricultural ecosystems. However, in intensive citrus cultivation systems, the impact of heavy fertilizer inputs on the microbially driven carbon pump and its contribution to SOC turnover and accumulation remains unclear. In citrus cultivation, the composition of soil microbial communities undergoes significant changes with increasing planting years (Zeng et al., 2022a; Zeng et al., 2022b), which may lead to substantial variations in their contribution to organic carbon. Previous studies had reported that citrus planted duration had declined bacterial diversity and suppressed the activity of phosphate-solubilizing bacteria (Zeng et al., 2022a; Zeng et al., 2024). These variations in the microbial community would alter the decomposition of soil organic matter and the pathways of SOC turnover. In addition, citrus cultivation with over fertilization resulted in a decline in soil pH and accumulation in soil available nitrogen and phosphorus (Zeng et al., 2022b), which would affect the microbial activity and soil microbial carbon use efficiency. Therefore, long-term citrus plantation would change the content and composition of microbial residue carbon, influencing the accumulation and transformation of SOC.

Currently, the quantification of microbial carbon in soil is primarily achieved through the measurement of specific biomarkers, such as amino sugar (Glaser et al., 2004; Joergensen, 2018). Amino sugar biomarkers are useful for characterizing the relative abundance of organic matter derived from various organisms and tissues within total SOC, as well as for tracing its transformation processes (Liang et al., 2019). The meta-analysis of amino sugar data revealed that the contribution of microbial residue carbon to SOC in the 0–20 cm soil layer varied across different land types, ranging from 33 to 62%, with farmland > grassland > woodland (Liang et al., 2019; Wang B. et al., 2021). Some studies have shown that microorganisms are a key source of SOC and play a central role in regulating its storage through microbial carbon pumps (Liang et al., 2017; Liang et al., 2019; Liang et al., 2020; Zhu et al., 2020; Deng and Liang, 2022; Xu et al., 2022). However, while the operation and efficiency of microbial carbon pumps are known to be strongly influenced by plant inputs (Chen et al., 2023; Feng and Wang, 2023), the temporal changes in these effects remain poorly understood.

To address this knowledge gap, this study would focus on analyzing amino sugars in the soil to explore changes in the sources of organic carbon under intensive cultivation with different durations. It aims to explore the impact of different citrus planting years on microbial residue carbon content and its contribution to SOC. The hypothesis is that with the increase in planting years, the content of microbial residue carbon in the soil would gradually decline, and its contribution mechanism to SOC may also change because of the decline in bacterial diversity (Zeng et al., 2024). Specifically, the research hypotheses are: (1) As the planting years of citrus increase, the content of microbial residue carbon (bacterial and fungal) in the soil would decrease; (2) The contribution of microbial residue carbon to SOC would decrease with increasing planting years; (3) There would be significant differences in the contribution of different types of microbial residue carbon to SOC, with fungal residue carbon contributing more to SOC than bacterial residue carbon as suggested in a global study. Varying planting years offer valuable insights into how the soil microbial carbon pump contributes to SOC sequestration and how it responds to citrus cultivation.

## 2 Materials and methods

### 2.1 Study site and soil sampling

This study focused on citrus orchards of varying planting ages in Shuitianba Township, Zigui County, located in the Three Gorges Reservoir Area. This region has a citrus cultivation history spanning several hundred years and is now a major navel orange production area. It has developed a streamlined operation system for breeding, grafting, planting, fertilization, harvesting, and sales. The management and fertilization practices in these orchards are consistent, with a circular fertilization approach involving 437.1 kg/hm^2^ of nitrogen (N), 178.8 kg/hm^2^ of phosphorus pentoxide (P₂O₅), and 178.8 kg/hm^2^ of potassium oxide (K₂O) (Zeng et al., 2022a). The soil is classified as purple soil, developed from purple sandstone. The region experiences a typical subtropical monsoon climate, with an average annual temperature of 18.2°C and an average annual precipitation of 940.3 mm (Zeng et al., 2022b). Currently, all available land in the region has been converted into citrus orchards.

To assess the long-term effects of citrus cultivation on SOC, this study selected orchards with different planting durations (10, 20, and 30 years). Additionally, to evaluate the potential impact of newly developed orchards on SOC, soil samples were collected from adjacent natural forests, predominantly composed of *Platycladus orientalis*. Due to the high economic value of citrus, more natural forests are gradually being converted into orchards. By comparing the natural forest land with the citrus orchards, this study aims to assess the potential impact of newly developed citrus orchards on SOC.

Soil samples were collected in September 2020. The citrus orchards were planted with Navel oranges (Newhall variety), and three orchards were selected for each planting year. In each orchard, 15 trees with similar growth were randomly chosen for sampling. Soil samples were taken from the drip line of each tree, avoiding fertilization pits. After removing surface debris, soil samples were collected from the 0–20 cm layer at the four corners surrounding each of the 15 trees within each orchard. The samples from each orchard were then combined into a composite sample. Stones, roots, dead branches, and leaves were removed, and the mixed samples were sieved through a 2 mm nylon mesh to ensure homogeneity. The soil collection in the natural woodland is the same as that of citrus.

Soil sampling in the adjacent natural forests followed the same procedure as in the citrus orchards. After removing surface litter, soil was collected from the 0–20 cm layer at multiple points and thoroughly mixed. Approximately 20 g of fresh soil was placed in a sterile bag and stored in an ice box for microbial analysis. Another 100 g of fresh soil was placed in a plastic bag for analysis of available nutrients, such as nitrate nitrogen and ammonium nitrogen. The remaining soils were air-dried at room temperature, away from direct sunlight, for subsequent analysis of SOC, organic carbon components, pH, and other physicochemical properties.

### 2.2 Soil analysis

#### 2.2.1 Soil physicochemical properties

Soil pH was determined by extracting soil using a 1:2.5 soil-to-water mass ratio and measuring it with a pH meter. Ammonium nitrogen (NH₄^+^-N) in the soil was extracted using 2 mol/KCl solution and quantified by the indophenol blue spectrophotometric method (Keeney and Nelson, 1982). Nitrate nitrogen (NO₃^−^-N) was also extracted using 2 mol//L KCl solution and determined by ultraviolet spectrophotometry. SOC was determined using the Walkley-Black method, as described in previous studies (Walkley and Black, 1934; Marriott and Wander, 2006). Soil available phosphorus (AVP) was extracted with 0.5 M sodium bicarbonate (NaHCO₃) solution at pH 8.5 (Olsen, 1954). After filtration, measure the phosphorus concentration in the extract using the molybdenum blue colorimetric method. Dissolved organic carbon (DOC) represents the fraction of organic carbon in the soil solution that is easily accessible for microbial activity. DOC in soil is extracted using a water-to-soil ratio of 1:10, and the extract is then analyzed using a total organic carbon analyzer.

#### 2.2.2 Determination of soil MAOC and POC

Mineral-associated organic carbon (MAOC) and particulate organic carbon (POC) contents were determined using the wet sieving method (Marriott and Wander, 2006; Chen et al., 2020; Lugato et al., 2021). A 20 g air-dried soil sample was weighed, and 100 mL of 5 g/L sodium hexametaphosphate solution was added. The mixture was shaken at a low speed for 18 h. The soil mixture was then wet-sieved using a 53 μm sieve: the portion passing through the sieve was designated as the MAOC solution, while the retained fraction represented the POC component. Both fractions were collected, dried in an oven at 65°C to a constant weight, and ground. The ground samples were passed through a 100-mesh sieve (0.149 mm), and the carbon content in both components was determined using the Walkley-Black method (Marriott and Wander, 2006; Walkley and Black, 1934).

#### 2.2.3 Determination of soil GRSPs

Soil glomalin-related soil proteins (GRSPs) were measured using the Bradford assay (Wright and Upadhyaya, 1996; Wright and Upadhyaya, 1998). One gram of soil sample was weighed into a centrifuge tube, and 8 mL of 0.05 mol/L sodium citrate solution was added. A blank control sample was prepared concurrently. The samples were sealed, mixed, and autoclaved at 121°C and 100 kPa for 60 min. After pressure release and cooling, the samples were centrifuged at 10000 r/min for 3 min, and the supernatant was transferred to a 50 mL centrifuge tube. The extraction was repeated until the supernatant became nearly colorless. A 0.5 mL aliquot of the centrifuged supernatant was transferred into a 10 mL centrifuge tube, and 5 mL of Coomassie Brilliant Blue dye was added. The mixture was shaken well, left to stand for 3 min, and the absorbance was measured at 595 nm using a 10 mm optical path cuvette.

A bovine serum albumin (BSA) stock solution (100 μg/mL) was prepared, and aliquots of 0, 20, 40, 80, 120, 160, and 200 μL were pipetted to obtain BSA concentrations of 0, 2, 4, 8, 12, 16, and 20 μg/mL, respectively. A 0.5 mL aliquot of each standard solution was transferred into a 10 mL centrifuge tube, and 5 mL of Coomassie Brilliant Blue dye was added and mixed well. After standing for 3 min, the absorbance was measured at 595 nm. The standard curve was plotted using protein concentration as the x-axis and absorbance as the y-axis.

#### 2.2.4 Determination of soil amino sugars

The extraction and determination of soil amino sugars were performed using the sugar esterification-derivatization method followed by gas chromatography (GC) (Zhang and Amelung, 1996). Soil samples with nitrogen content greater than 0.3 mg were weighed, and 10 mL of 6 mol/hydrochloric acid was added. The mixture was hydrolyzed in an oven at 105°C for 8 h. After hydrolysis, 100 μL of inositol solution (1 mg/mL) was added as an internal standard. The hydrolyzed product and remaining soil were transferred into a round-bottom flask using filter paper, and evaporated to dryness under reduced pressure at 53°C. The dried residue was dissolved in a small amount of distilled water, and the pH was adjusted to 6.6–6.8 using KOH and dilute HCl. The solution was centrifuged at 3000 rpm for 10 min, and the supernatant was transferred for freeze-drying. Once freeze-dried, the residue was dissolved in anhydrous methanol and transferred to a derivatization flask. The sample was nitrogen-blown to dryness at 45°C, re-dissolved in 1 mL of pure water, and 100 μL of N-methylglucosamine (1 mg/mL) was added as a second internal standard. The sample was frozen and freeze-dried again.

To derivatize, 300 μL of derivatizing reagent (a 4:1 mixture of 32 mg/mL hydroxylamine hydrochloride and 40 mg/mL 4-dimethylaminopyridine) was added to the freeze-dried sample. The mixture was vortexed and heated in a water bath at 75–80°C for 35 min. Afterward, 1 mL of acetic anhydride was added and heated at 75–80°C for another 60 min. The solution was then treated with 1.5 mL of dichloromethane, vortexed, and 1 mL of 1 mol·L^−1^ hydrochloric acid was added. After vortexing, the sample was separated into organic and aqueous phases. The upper aqueous layer was discarded, and the process was repeated three times with pure water to remove any residual inorganic phase. The remaining organic phase was nitrogen-blown to dryness at 45°C. The dried residue was then dissolved in 200 μL of a 1:1 mixture of ethyl acetate and n-hexane, and transferred to a 1.5 mL headspace vial for gas chromatography (GC) analysis.

The area of the peaks was used to quantify four amino sugars: GluN, ManN, GalN, and MurA. Fungal and bacterial residue carbon were estimated based on the amounts of GluN and MurA, using the following Equations 1 and 2 (Joergensen, 2018; Liang et al., 2019):


(1) FNC=(GluN/179.17–2×MurA/251.23)×179.17×9

(2) BNC=MurA×45

Where FNC is fungal residue carbon; BNC is bacterial residue carbon; 179.17 is the molecular weight of GluN; 251.23 is the molecular weight of MurA; 45 is the conversion factor from MurA to bacterial residue carbon; and 9 is the conversion factor from GluN to fungal residue carbon.

### 2.3 Data statistical analysis

The contents of MNC, BNC, and FNC in citrus soils of different planting years, along with their contributions to SOC, were analyzed using one-way analysis of variance (ANOVA) with a significance level of *p* < 0.05, conducted in IBM SPSS 27.0 (SPSS Inc. Chicago, IL, USA). Prior to ANOVA, homogeneity of variance was tested, and non-parametric tests were applied to data that did not follow a normal distribution. Pearson correlation analysis was used to assess the relationship between basic soil properties and various organic carbon components. To quantitatively assess the impact of each potential environmental factor on microbial residue carbon and SOC, a multiple regression model was used in R 4.3.2. Based on a multiple linear model, the relative importance (RI) of each significant variable was calculated using the lmg function from the relaimpo package (Grömping, 2007; Zeng et al., 2019). This method provides a measure of the contribution of each variable, helping to quantify and rank the influence of different environmental factors on microbial residue carbon. The random forest model assessed the influence of environmental factors on SOC, BNC, and FNC.

## 3 Results

### 3.1 Soil properties at different planted ages

The duration of citrus cultivation significantly affects soil pH, nitrate nitrogen, available phosphorus, ammonium nitrogen, organic carbon, and organic carbon components (*p* < 0.05) (Table 1). SOC showed a gradual increase, rising from 9.10 g/kg at 10 years of cultivation to 18.33 g/kg at 30 years. However, dissolved organic carbon (DOC) showed no significant differences across soils of different planting durations. POC, MAOC, and GRSP all exhibited an increasing trend with longer cultivation periods, leading to a significant increase in SOC. In natural forest soils, POC content was higher than MAOC, while in citrus soils, MAOC content exceeded POC.

**Table 1:** Soil properties and organic carbon fractions at different planted ages.

Soil properties	NF	10a	20a	30a
pH	6.58 ± 0.02b	7.66 ± 0.11a	4.88 ± 0.20c	3.69 ± 0.17d
NH + 4-N (mg/kg)	20.47 ± 0.29c	11.93 ± 0.36d	30.78 ± 3.19b	38.48 ± 5.48a
NO- 3-N (mg/kg)	21.47 ± 0.28c	6.86 ± 0.31d	29.15 ± 0.97b	40.93 ± 0.61a
AVP (mg/kg)	5.00 ± 0.26d	72 ± 0.21c	132 ± 0.50b	188 ± 0.40a
DOC (g/kg)	155 ± 15a	97 ± 14b	93 ± 8.0b	93 ± 14b
SOC (g/kg)	17.27 ± 0.11b	9.10 ± 0.12d	13.69 ± 0.09c	18.33 ± 0.54a
POC (g/kg)	10.07 ± 1.24a	2.36 ± 0.41d	3.13 ± 0.19c	7.33 ± 3.02b
MAOC (g/kg)	6.79 ± 0.15b	4.45 ± 0.27c	11.16 ± 0.8a	10.62 ± 0.68a
GRSP (g/kg)	2.04 ± 0.03a	0.98 ± 0.03c	1.87 ± 0.07b	1.86 ± 0.08b

NH+ 4-N, Ammonium nitrogen; NO- 3-N, Nitrate nitrogen; AVP, Soil available phosphorus; DOC, Dissolved organic carbon; SOC, Soil organic carbon; MAOC, mineral-associated organic carbon; POC, Particulate organic carbon; GRSP, Total glomalin-related soil proteins. NF, Natural forest; 10a, 10-year-old citrus orchards; 20a, 20-year-old citrus orchards; 30a, 30-year-old citrus orchards. Different lowercase letters indicate significant differences between natural forest and citrus soils.

### 3.2 Soil amino sugar content at different planted ages

**Figure 1:**
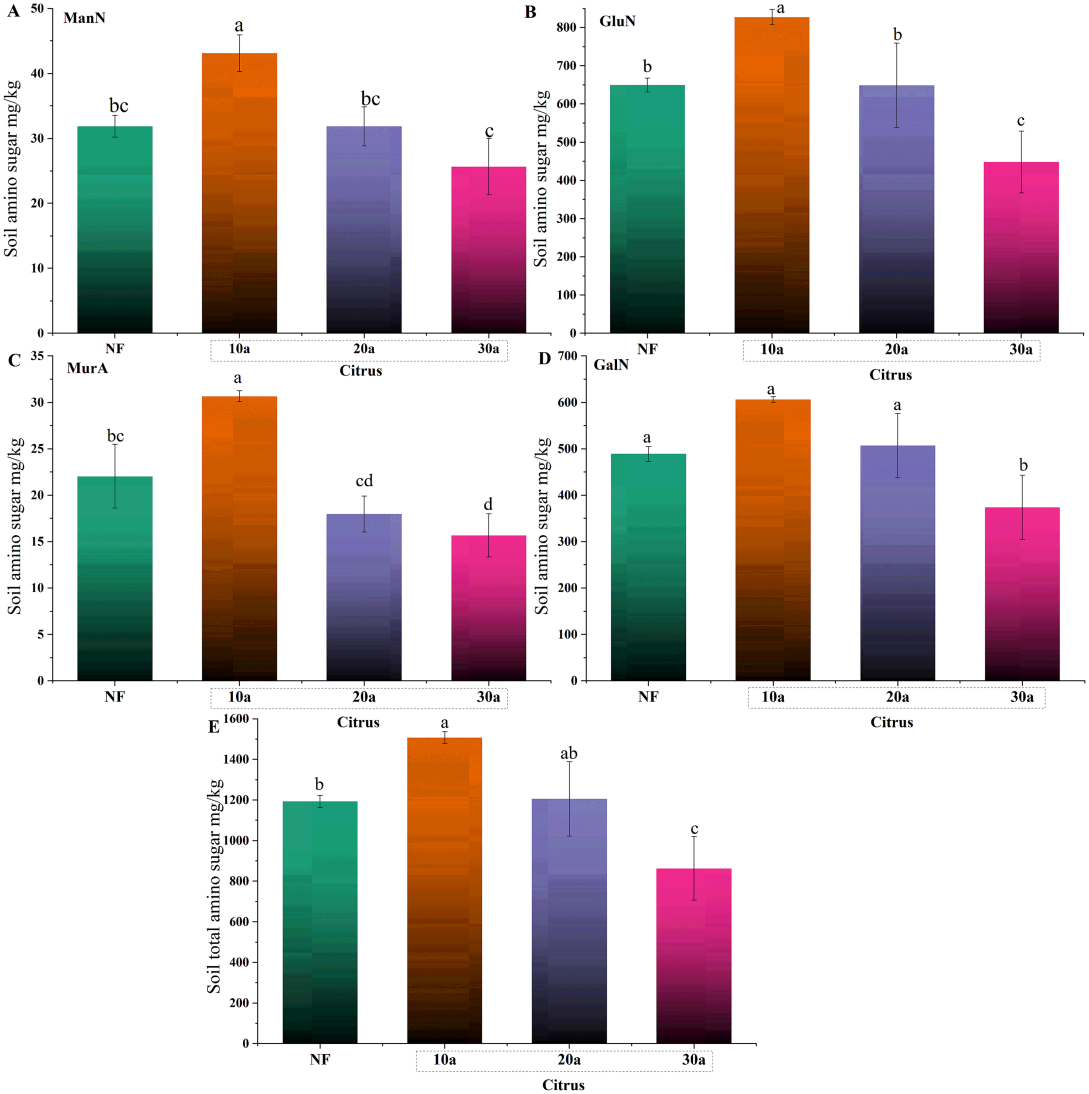
Changes in soil amino sugar content during intensive citrus cultivation. **(A)** Mannosamine (ManN), **(B)** glucosamine (GluN), **(C)** muramic acid (MurA), and **(D)** galactosamine (GalN). NF, natural forest; 10a, 10-year-old citrus orchards; 20a, 20-year-old citrus orchards; 30a, 30-year-old citrus orchards. Different lowercase letters indicate significant differences between natural forest and citrus soils (*p* < 0.05).

The contents of four amino sugars (GluN, GalN, ManN, MurA) showed a significant decreasing trend with increasing planting years (*p* < 0.05) (Figure 1). Compared to the 10-year-old citrus soil, the content of GluN in the 30-year-old citrus soil decreased by 45.83%, GalN by 38.44%, ManN by 40.6%, and MurA by 48.92%. In comparison to citrus soil, the amino sugar content in natural forest soil followed the trend of 10a > NF > 30a, with no significant difference observed between the 20-year-old citrus soil and natural forest soil (*p* > 0.05).

### 3.3 Soil microbial necromass carbon and their contribution to SOC at different planted ages

**Figure 2:**
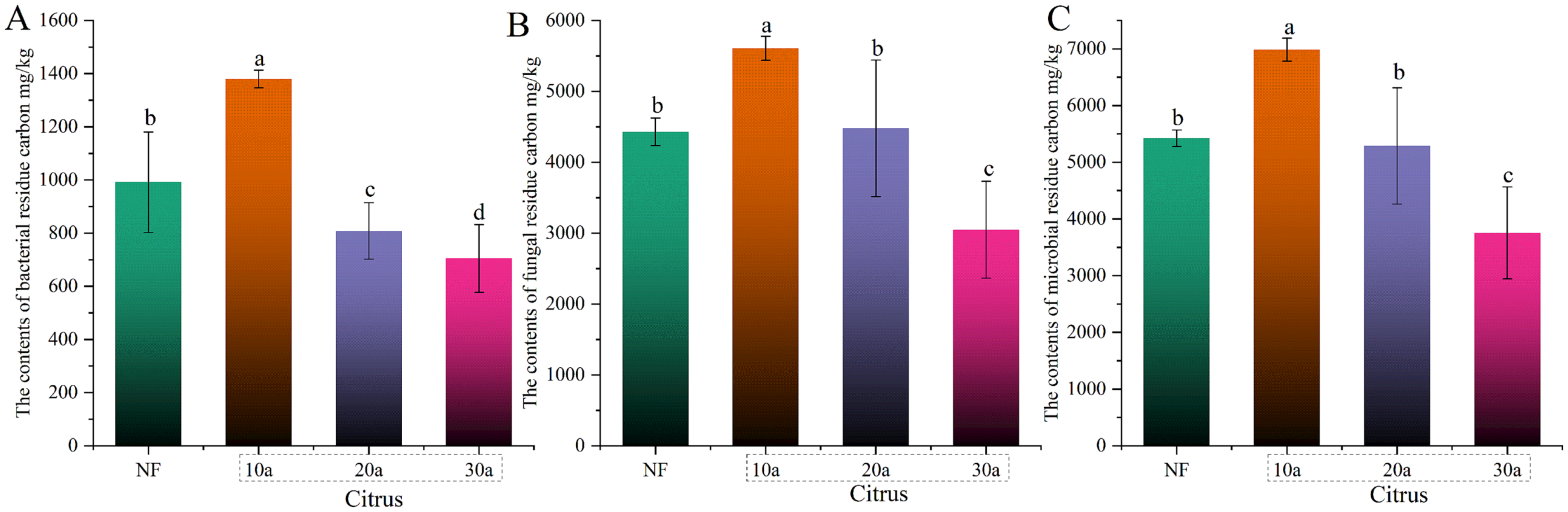
Changes in microbial residue carbon during intensive citrus cultivation. **(A)** Microbial residue carbon (MNC), **(B)** fungal residue carbon (FNC), and **(C)** bacterial residue carbon (BNC). NF, natural forest; 10a, 10-year-old citrus orchards; 20a, 20-year-old citrus orchards; 30a, 30-year-old citrus orchards. Different lowercase letters indicate significant differences between natural forest and citrus soils.

During intensive cultivation, the contents of MNC, FNC, and BNC significantly decreased with increasing planting years (*p* < 0.05) (Figure 2). Compared to the 10-year-old citrus soil, MNC, FNC, and BNC contents in the 30-year-old citrus soil decreased by 46.27, 45.61, and 48.91%, respectively. The MNC and FNC contents in natural forest soil showed no significant differences compared to the 20-year-old citrus soil (*p* > 0.05), but were significantly lower than in the 10-year-old citrus soil and significantly higher than in the 30-year-old citrus soil (*p* < 0.05).

**Figure 3:**
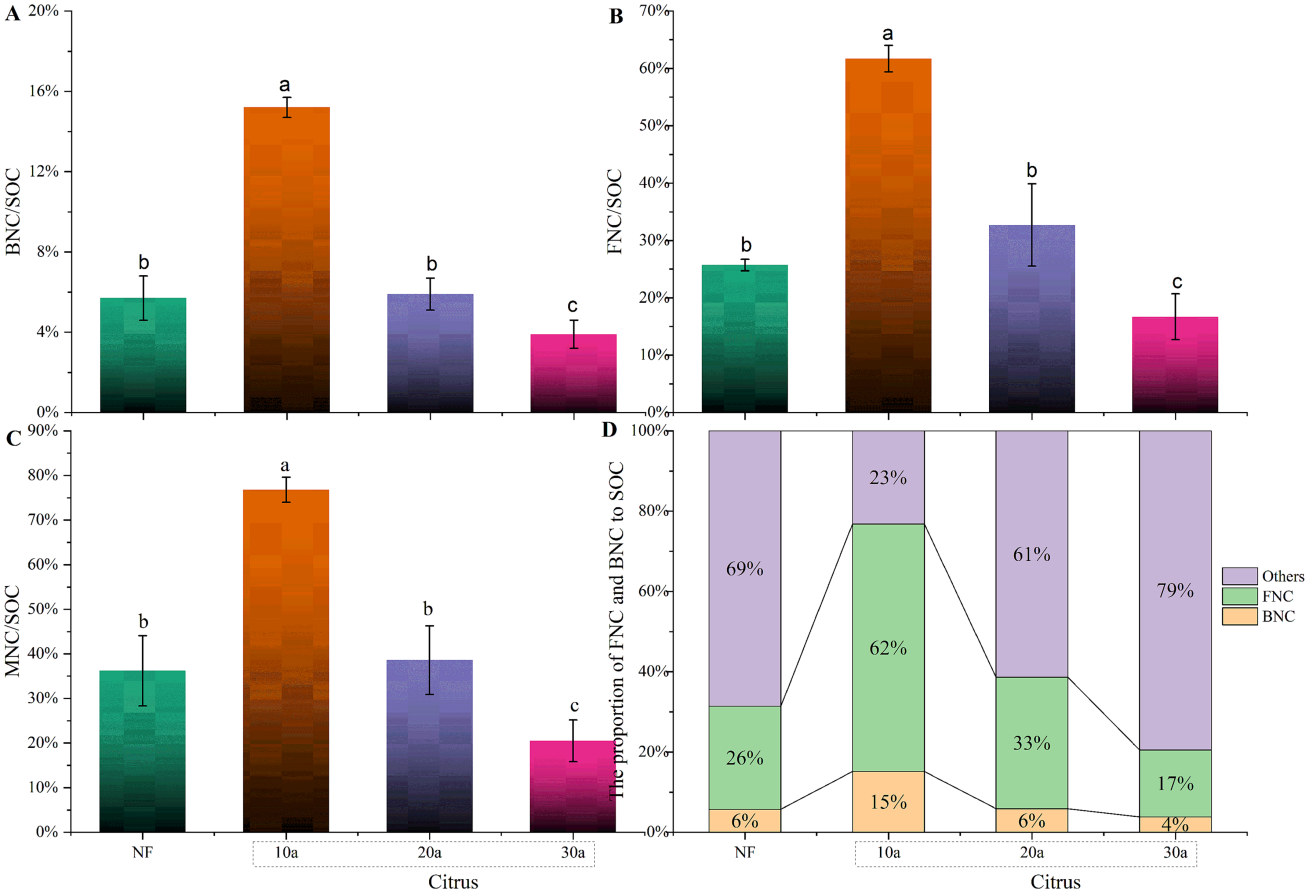
Proportion of organic carbon derived from different sources in the total organic carbon content of citrus soils at various planting years. **(A)** Proportion of BNC to SOC. **(B)** Proportion of FNC to SOC. **(C)** Proportion of MNC to SOC. **(D)** Proportion of BNC and FNC to SOC. NF, natural forest; 10a, 10-year-old citrus orchards; 20a, 20-year-old citrus orchards; 30a, 30-year-old citrus orchards. Different lowercase letters indicate significant differences between natural forest and citrus soils.

The proportion of microbial residue carbon to SOC significantly decreased with increasing planting years (*p* < 0.05), from 76.82 ± 2.84% in the 10-year-old citrus soil to 20.54 ± 4.70% in the 30-year-old citrus soil (Figure 3). The ratio of MNC/SOC in the soil followed the order of 10a > NF > 30a. There was no significant difference in MNC/SOC between the natural forest soil and the 20-year-old citrus soil (*p* > 0.05). The contribution of bacterial and fungal residue carbon to SOC exhibited similar trends to that of total microbial residue carbon, significantly decreasing with increasing citrus planting years, with reductions of 74.60 and 72.94%, respectively, from 10a to 30a. In natural forest soil, the bacterial and fungal residue contributions were both higher than those in the 30-year-old citrus soil but lower than those in the 10-year-old citrus soil.

### 3.4 Determinants of microbial necromass carbon accumulation

To quantify the potential impact of various environmental factors on soil microbial residue carbon, a multiple regression model was used to assess the relative contribution of key environmental factors. The results indicated that the model explained 97.15% of the variation in soil microbial residue carbon. Among these, soil pH, NO_3_^−^-N, MAOC, and GRSP were identified as the main factors influencing microbial residues in the soil (*p* < 0.01), with their relative importance being 0.31, 0.30, 0.21, and 0.18, respectively (Table 2).

**Table 2:** Quantifying the contribution of environmental factors to soil microbial residue carbon using multiple regression models.

Factors	Estimate	Std.Error	*t* value	*p*	RI
(Intercept)	−28,740	5,996	−4.79	0.0020	
MAOC	889	134	6.65	0.0003	0.21
pH	4,322	716	6.03	0.0005	0.31
NO_3_^−^-N	345	79	4.39	0.0032	0.30
GRSP	−3,774	639	−5.91	0.0006	0.18

RI, Relative importance; MAOC, Mineral-bound organic carbon; NO_3_^−^-N, Nitrate nitrogen; GRSP, Total glomalin-related soil proteins.

**Figure 4:**
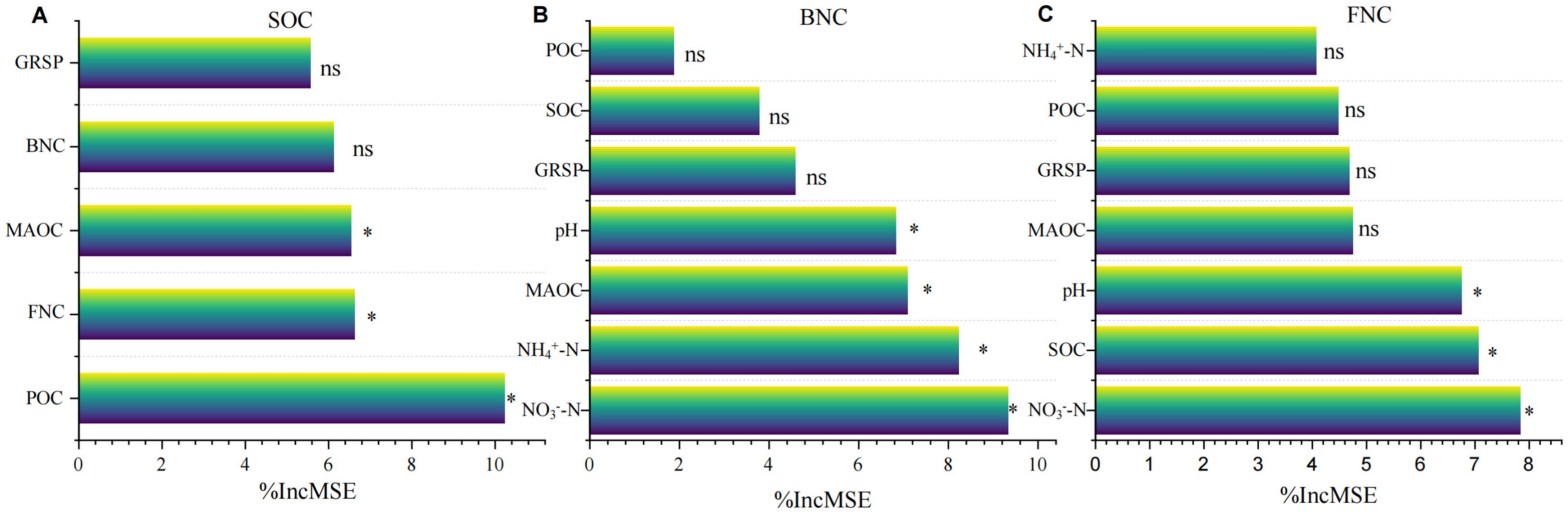
The effects of environmental factors on **(A)** SOC, **(B)** bacterial nitrogen content (BNC), and **(C)** fungal nitrogen content (FNC). Bars marked with “*” represent variables that are significant at the *p* < 0.05 level.

Correlation analysis showed a significant positive correlation between soil pH and MNC, BNC, FNC, and a significant negative correlation with NO_3_^−^-N, NH_4_^+^-N, SOC, MAOC, and GRSP. There was no significant correlation between pH and DOC or POC. Random forest model analysis indicated that POC, MAOC, and FNC were the most important contributing factors to SOC. The main factors affecting FNC were NO_3_^−^-N, SOC, and pH, while the main factors influencing BNC were NO_3_^−^-N, NH_4_^+^-N, MAOC, and pH (Figure 4, *p <* 0.05).

## 4 Discussion

### 4.1 Decline in microbial necromass carbon along with citrus planted years

We found that microbial necromass carbon decreased with increasing years of plantation, from 10 to 30 years, consistent with our hypothesis. This finding suggested that microbes contribute less necromass in soils under long-term citrus plantations. Across all soils, the contribution of fungal necromass carbon to SOC was greater than that of bacterial necromass carbon, suggesting that fungi had a more prominent role in soil organic carbon accumulation (Shao et al., 2021; Zhao et al., 2023; Yu et al., 2024). Fungi predominantly contributed to microbial necromass, likely due to their ability to decompose complex organic compounds such as cellulose, hemicellulose, and lignin. Even in low-oxygen environments, such as marine sediments (Niggemann and Schubert, 2006) and paddy soils (Amelung, 2001), fungal necromass carbon continued to play a dominant role. Previous studies had shown that the turnover rates of fungal and bacterial phospholipid biomass are similar (Zhang et al., 2019), but fungi could utilize a broader range of recalcitrant compounds as energy sources compared to bacteria (Bahram et al., 2018). This competitive advantage of fungi resulted in a greater contribution of fungal necromass to the total microbial necromass carbon in soil after biomass turnover. Our findings also supported this, as fungal necromass carbon was consistently higher than bacterial necromass carbon in soils with different citrus plantation ages. Furthermore, the higher carbon-to-nitrogen ratio of fungal necromass makes bacterial necromass the preferred source for microbial reutilization. This prioritization serves as a more efficient nutrient acquisition strategy, leading to lower bacterial necromass retention in the soil. Compared to bacterial residues, fungal residues are larger, possess thicker cell walls, and readily form complex macromolecular aggregates, enhancing their stability (Schweigert et al., 2015; Adamczyk et al., 2019). Therefore, fungal necromass played a significantly greater role in SOC accumulation than bacterial necromass.

Although long-term citrus cultivation had led to negative effects such as soil acidification and reduced microbial diversity (Zeng et al., 2022b, 2024), the first 10 years of citrus cultivation were beneficial in terms of microbial necromass. During this period, both the content of microbial necromass and its contribution to SOC were favorable, with its contribution reaching as high as 77%, significantly higher than that in the adjacent natural forest. Therefore, in the later stages of citrus cultivation, attention should be given to the negative effects of soil acidification on microbial contributions to organic carbon.

### 4.2 Factors affecting microbial necromass carbon and its contribution to SOC

Our study showed that soil pH was an important environmental factor influencing microbial necromass carbon. Microbial necromass carbon in soil was primarily affected by microbial activity, microbial carbon use efficiency, and the life strategies of microbial communities (Shao et al., 2021). In this study, soil acidification was significant, with soil pH dropping by nearly 4 units from citrus plantations aged 10 years to those aged 30 years. The acidification of citrus soils directly reduced bacterial diversity, altered microbial community composition and activity (Fierer and Jackson, 2006; Malik et al., 2018; Zeng et al., 2024), which in turn affected the production of microbial necromass. These mechanisms highlighted the importance of microbial ecophysiological controls on soil organic matter accumulation in soils with high pH (Malik et al., 2018). In near-neutral pH soils, microbes efficiently utilized substrates to synthesize biomass, resulting in greater carbon storage potential (Malik et al., 2018), which also explained why microbial necromass carbon in citrus soils aged 10 years contributed significantly more to SOC than in soils aged 20 or 30 years. In low pH soils, however, intensification required additional non-microbial carbon inputs, such as plant inputs or soil acidification amendments, to increase SOC content. In this study, while longer citrus cultivation periods promoted organic carbon accumulation, the microbial contribution to organic carbon decreased.

The contribution of microbial necromass to SOC increased with soil pH, mainly because pH affected the accumulation of microbial necromass. High soil acidity reduced the accumulation of fungal and bacterial necromass. This might be due to (i) lower pH inhibiting microbial growth, particularly bacteria (Rousk et al., 2009), which in turn reduced microbial biomass and necromass accumulation, and (ii) a reduction in microbial carbon use efficiency (CUE) (Shi et al., 2024) and microbial turnover rates (Wang C. et al., 2021) at lower pH. A large-scale study reported a marginal positive correlation between microbial CUE and soil pH, and that microbial turnover rates increased with higher pH (Wang C. et al., 2021). In low pH soils, microbes allocated more carbon to respiration processes and less to biomass formation, leading to lower microbial necromass accumulation. Additionally, microbial necromass in high pH soils was less prone to decomposition; for example, chitin decomposition from fungal necromass responded negatively to soil pH (Hu et al., 2020). Therefore, lower pH soils had relative lower soil microbial necromass.

Furthermore, the contribution of microbial necromass to SOC was also influenced by nitrogen availability. In agricultural ecosystems, excessive nitrogen fertilization led to soil acidification, reduces microbial diversity and carbon use efficiency, and decreases microbial necromass production. A large-scale study found that microbial biomass turnover rates increased with soil pH (Wang C. et al., 2021); therefore, soil acidification could reduce necromass accumulation. However, moderate nitrogen fertilization could promote microbial growth, enhanced carbon use efficiency, and stimulated substrate synthesis, thus increasing microbial necromass and its contribution to organic carbon (Ni et al., 2021). For example, in soils of 10-year-old citrus plantations, both bacterial and fungal necromass were significantly higher than those in the adjacent natural forest. This suggested that appropriate nitrogen fertilization during the early stages of citrus cultivation could effectively stimulate microbial substrate synthesis, leading to higher microbial necromass carbon production. Therefore, reducing excessive nitrogen fertilization in the later stages of citrus cultivation was a critical issue that needs to be addressed to mitigate soil acidification and microbial functional degradation in citrus soils.

### 4.3 Implications and prospects of MNC in intensive citrus orchards

Our study was based on data obtained from a long-term experimental series. The results showed a significant decreasing trend in soil microbial necromass carbon content and its contribution to SOC. This emphasized that citrus cultivation was weakening the contribution of soil microorganisms to SOC, even though SOC was increasing, indicating that other sources of organic carbon, such as plant-derived carbon, might be increased. Despite the significant decrease in the proportion of microbial necromass carbon in soils from 30-year-old citrus plantations compared to forest soils and 10-year-old citrus soils, its contribution remained noteworthy. Therefore, long-term citrus cultivation not only led to the loss of soil microbial diversity, resulting in reduced microbial necromass yields, but also decreased the contribution of microbial necromass to SOC.

Additionally, we used amino sugars to calculate the contribution of microorganisms to total organic carbon, which had some limitations. First, the content of glucosamine in fungi and muramic acid in bacteria from culture experiments differed from that of actual soil microbes (Joergensen, 2018). Second, the ratio of Gram-positive to Gram-negative bacteria varied across different soil types, directly influencing the contribution of bacterial necromass to organic carbon. Amino sugars only represented a portion of microbial necromass, and they could also be metabolized by microorganisms. Although the proportion of amino sugars metabolized by microorganisms is low, it may still cause some bias. Therefore, future research should focus on microbial necromass accumulation coefficients. Existing studies have shown that there are differences across regions and soil layers, with surface soils contributing less than deeper soils (Wang B. et al., 2021). This study only focused on the changes in microbial necromass and its contribution to organic carbon in surface soils and did not consider deeper soils. Future studies should give more attention to the regions reached by citrus root systems, as rhizosphere microorganisms may also affect deep SOC. The rhizosphere is a microbial “hotspot” and a key area for the operation of the soil microbial carbon pump, so future research should distinguish between rhizosphere and non-rhizosphere soils.

## 5 Conclusion

As the duration of citrus cultivation increased, the content of MNC and its contribution to SOC in citrus soils gradually declined. In all soils, the contribution of FNC to SOC become dominant, significantly surpassing that of BNC. A significant negative correlation between microbial necromass carbon and SOC suggested a weakening of the microbial carbon pump in citrus soils, with an increasing reliance on carbon from other sources. MNC in citrus soils was primarily influenced by factors such as soil pH, nitrate nitrogen, ammonium nitrogen, and mineral-bound carbon. Specifically, lower pH reduces the accumulation of soil microbial carbon and reduces the contribution of microbial carbon to SOC. The accumulation of nitrate and ammonium nitrogen in citrus soils further acidified the soil, which reduced pH and impaired microbial carbon pump efficiency, thus limiting microbial necromass carbon accumulation. In summary, the buildup of high levels of nitrate and ammonium nitrogen under citrus cultivation led to soil acidification, which diminished the microbial contribution to organic carbon. To mitigate this, strategies aimed at enhancing microbial diversity to stimulate the microbial carbon pump, as well as increasing microbial necromass carbon, could effectively boost the SOC pool in citrus soils. The findings of this study provided valuable insights into the impacts of long-term citrus plantation on soil microbial necromass carbon.

## Data Availability

The raw data supporting the conclusions of this article will be made available by the authors, without undue reservation.
